# Candida auris on Apples: Diversity and Clinical Significance

**DOI:** 10.1128/mbio.00518-22

**Published:** 2022-03-31

**Authors:** Anamika Yadav, Kusum Jain, Yue Wang, Kalpana Pawar, Hardeep Kaur, Krishan Kumar Sharma, Vandana Tripathy, Ashutosh Singh, Jianping Xu, Anuradha Chowdhary

**Affiliations:** a Medical Mycology Unit, Department of Microbiology, Vallabhbhai Patel Chest Institute, University of Delhi, Delhi, India; b Department of Zoology, Ramjas College, University of Delhi, Delhi, India; c Department of Biology, McMaster University, Hamilton, Ontario, Canada; d All India Network Project on Pesticide Residues, ICAR-Indian Agricultural Research Institute, New Delhi, India; University of Toronto; Duke University; Centers for Disease Control and Prevention

**Keywords:** *C. auris* ecology, natural environment, fungicides, dimethyl inhibitors, cross-resistance, agriculture azoles

## Abstract

Candida auris is a multidrug-resistant nosocomial fungal pathogen. While the marine environment was recently identified as a natural niche for C. auris, the environment(s) that might have contributed to the development and spread of antifungal resistance in C. auris remains a mystery. Because stored fruits are often treated with fungicides to prevent postharvest spoilage, we hypothesized that stored fruits could serve as a possible selective force for and a transmission reservoir of antifungal-resistant isolates of pathogenic yeasts, including C. auris. To test this hypothesis, we screened fruits to study the diversity of pathogenic yeasts and their antifungal susceptibility profiles. Among the 62 screened apples, the surfaces of 8 were positive for C. auris, and all were stored apples. Whole-genome sequencing (WGS) showed that C. auris strains from apples were genetically diverse and exhibited broad phylogenetic distribution among the subclades within clade I. Interestingly, strains from apples had closely related strains from other sources in India, including from patients, hospitals, and marine environments, and from clinical strains from other parts of the world. A broad range of fungicides, including dimethyl inhibitors (DMIs), were detected in stored apples, and all C. auris isolates exhibited reduced sensitivity to DMIs. Interestingly, C. auris was not isolated from freshly picked apples. Together, the results suggest a potentially complex ecology for C. auris with agriculture fungicide application on stored fruits as a significant selective force for drug resistance in clinics.

## INTRODUCTION

Candida auris is a fungal pathogen and a serious threat to global health (1). Since 2009, C. auris has caused an escalating number of health care-associated outbreaks. This recently emerged pathogen exhibits high rates of drug resistance and high transmissibility within health care facilities, causing a significant challenge for treating infected patients and for eradicating the pathogen from health care settings (2). Since its first identification in 2009 in Japan, C. auris has independently emerged and/or spread to all populated continents, with several regions reporting clinical strains belonging to phylogenetically distant clades (3–5). Indeed, genomic analyses have revealed the near-simultaneous emergence of five distinct lineages across six continents, encompassing over 40 countries within the past ∼400 years (4, 6). A recent report about the isolation of C. auris from the marine environment in India suggested a potential natural niche for C. auris (7). Specifically, the study reported isolation of C. auris from the sandy beach and a tidal swamp in the tropical Andaman Islands, with several strains showing close genetic relationships to clinical isolates in mainland India. Interestingly, compared to the common feature of multidrug resistance among clinical strains, one C. auris strain from the tidal marsh was sensitive to all tested antifungal drugs. They hypothesized that the drug-susceptible C. auris strain from an aquatic habitat with no known human activity might represent an ancestral state of the pathogen which subsequently developed drug resistance during its adaptation to anthropogenic environments (7, 8). At present, the environments that might have contributed to the development and spread of antifungal resistance in C. auris remain a mystery.

In natural environments, yeasts are predominantly saprotrophs and have been found on many types of ecological niches where organic compounds are common, including soil, wood, litter, and the surfaces of leaves and fruits (9). For example, several species of yeasts were isolated from fruits in tropical and subtropical habitats, including opportunistic human pathogens Candida krusei, Candida orthopsilosis, Candida parapsilosis, Candida pelliculosa, and Candida tropicalis (10–12). Notably, Lo et al. found fluconazole-resistant C. tropicalis on the surface of fruits and with genotypes similar or identical to those infecting humans (11). Given that stored fruits are often treated with fungicides to prevent postharvest spoilage and to extend their shelf life, the results suggest that in addition to acting as an ecological niche for pathogenic yeasts, fruits could also be a possible selective force for and a transmission reservoir of antifungal-resistant isolates of human-pathogenic yeasts (13). However, so far, no isolate of C. auris has been reported from fruits.

In this study, we screened seasonal tropical and temperate fruits to study the ecological diversity of pathogenic yeasts with an emphasis on the isolation of C. auris. Colonies of C. auris were obtained from the surfaces of stored apples. The antifungal susceptibilities and whole-genome sequences of all C. auris isolates were obtained and compared with those of previously reported strains from India and other regions. Our results suggest that exposure to fungicides used for fruit storage could be a selective force for azole resistance in C. auris in clinical settings.

## RESULTS

### Yeast diversity on tropical and temperate fruits.

This study analyzed fruit samples collected from March 2020 to September 2021. We surveyed a total of 84 fruits representing nine fruit plant species, including seasonal tropical and temperate fruits collected from New Delhi, adjoining National Capital Region (NCR), and other regions of northern India. We focused on investigating yeasts on the fruits’ surfaces using the swabbing technique. A total of 144 yeast strains belonging to 22 species were isolated from the surface (epicarp) of fruits of the nine sampled species, with each fruit species containing at least one yeast species (Table 1). Different from the epicarp, no yeast was isolated from the endocarp tissue of any of the 84 fruits that we screened. Among the isolated yeast genera, the genus *Candida* predominated (73%) the surfaces of all the fruits investigated, with C. guilliermondii (20.8%) being the most common, followed by C. parapsilosis (15.9%), C. tropicalis (13%), and C. auris (11%).

**TABLE 1:** Distribution of yeast species (*n* = 22) isolated from nine fruit plant species (tropical and temperate)^*a*^

Slot no.	Species (no. of colonies)	Fruit(s) (common name)
1	Candida auris (*n* = 16)	*Malus domestica*^TEM^ (apple)
2	C. lusitaniae (*n* = 7)	M. domestica
3	C. parapsilosis (*n* = 23)^*b*^	M. domestica
4	Lodderomyces elongisporus (*n* = 13)^*b*^	M. domestica
5	C. carpophila (*n* = 1)	M. domestica
	*C. blankii* (*n* = 1)	M. domestica
	C. magnoliae (*n* = 1)	M. domestica
	C. rugosa (*n* = 1)	M. domestica
	C. albicans (*n* = 1)	M. domestica
	Issatechnika terricolis (*n* = 1)	M. domestica
	Hansempora uvarum (*n* = 1)	M. domestica
	H. vineac (*n* = 1)	M. domestica
6	C. tropicalis (*n* = 19)^*b*^	M. domestica, Cucumis melo^TRP^ (Melon), Mangifera indica^TRP^ (Mango), Pyrus pyrifolia^TEM^ (pear, kishtabahira, Asian pear), Punica granatum^TRP^ (pomegranate), Citrus sinensis^TRP^ (orange)
7	C. guilliermondii (*n* = 30)^*b*^	M. domestica, M. indica, Prunus bokharensis^TEM^ (plum, Bokhara plum)
8	C. caribbica (*n* = 2)	M. domestica, C. melo
9	Kodamea ohmeri (*n* = 16)^*b*^	M. domestica, *C. melo*
10	Trichosporan asahii (*n* = 1)	*C. melo*
	C. krusei (*n* = 1)	
11	Pichia manshurica (*n* = 1)	V. vinifera^TRP^ (grapes)
12	Merozyma ferinosa (*n* = 3)	P. pyrifolia
13	C. kefyr (*n* = 2)	Ananas comosus^TRP^ (pineapple), *P. granatum*
14	Aureobasidium pollulans^*c*^ (*n* = 2)	*M. indica*

a TRP; tropical fruit, TEM; temperate fruit.

b Two to six colonies per apple surface.

c Black yeast.

**C. auris on apple surfaces.** In this study, due to the broad cultivation of apples around the world and their year-long availability in our local markets, we focused on investigating yeasts from apples, with 62 of the 84 sampled fruits being apples. In addition, we focused on the two most common varieties of apples in India, Red Delicious and Royal Gala. These apples included both freshly harvested (*n* = 20) and stored apples (*n* = 42). The stored apples were purchased from local fruit vendors of Delhi and adjoining NCR between March and April in 2020 and between June and July in 2021. The fresh apples, all Red Delicious variety, were collected during the harvesting season (September 2021) from four apple orchards located in northern India, in the Solan and Kullu districts of Himachal Pradesh and in Srinagar city of Jammu and Kashmir. Two of the four orchards, one in Solan and the other in Kullu, used organic farming. In contrast, the orchard in Srinagar and the other orchard in Solan used nonorganic farming approaches.

Among the 62 apples, 8 (13%) yielded C. auris. A total of 16 C. auris colonies were recovered from surfaces of these eight apples (Tables 1 and 2). Candida auris was isolated from the surfaces of both the Red Delicious (*n* = 5) and the Royal Gala (*n* = 3) varieties. Interestingly, all 16 C. auris colonies were from stored apples purchased from the local fruit vendors. In contrast, only 1 of the 20 fresh apples collected from the four orchards resulted in a single yeast colony belonging to the species C. albicans. Overall, the surfaces of the eight C. auris-positive apples yielded 1 to 5 colonies each by surface swabbing. Interestingly, 4 of 8 apples positive for C. auris harbored only this yeast species, whereas the remaining 4 apples were co-colonized by C. parapsilosis, C. guilliermondii, and/or Kodamaea ohmeri. In contrast to the low yeast diversity of the 8 stored apples that were positive for C. auris, the 34 stored apples from local markets that were negative for C. auris had high yeast diversity. In total, these 34 apples harbored 16 species, including 11 spp. of *Candida* and 5 species in five other genera (Tables 1 and 2).

**TABLE 2:** Spectrum of yeast species from the surfaces of 62 apples and detection of fungicides

Slot no. of apples^*a*^	Date of collection	Apple variety	No. of apples positive for C. auris/total apples screened (no. of C. auris colonies)	co-colonizing yeast species	Fungicide detected (concn in ppm)		
					Category of fungicides	C. auris-positive apples	C. auris-negative apples
1	16 March 2020	Red Delicious	2/5 (*n* = 3)	C. parapsilosis	Not done	Not done	Not done
				C. guilliermondii			
2	15 April 2020	Royal Gala	1/9 (*n* = 5)		Not done	Not done	Not done
3	4 June 2021	Royal Gala	1/3 (*n* = 2)		Triazoles	Flusilazole^*b*^ (0.02)	Flusilazole^*b*^ (0.01–0.02)
						Sulfentrazone (0.019)	
					Diazoles	Carbendazim (0.07)	Carbendazim (0.04–0.19)
					Other fungicides	Kresoxim-methyl (0.05)	Kresoxim-methyl (0.05–0.18)
4	26 July 2021	Royal Gala	1/5 (*n* = 1)	C. guilliermondii	Triazoles	Flusilazole^*b*^ (0.02)	Flusilazole^*b*^ (0.02–0.09)
					Diazoles	Carbendazim (0.07)	Carbendazim (0.04–0.19)
					Other fungicides	Kresoxim-methyl (0.05)	Kresoxim-methyl (0.05–0.18)
5	26 July 2021	Red Delicious	1/5 (*n* = 2)		Triazoles	Sulfentrazone (0.019)	Sulfentrazone (0.013–0.016)
					Diazoles	Fludioxonil (0.01)	Fludioxonil (0.03)
						Pyroclostrobin (0.02)	Pyroclostrobin (0.01–0.02)
					Other fungicides	Boscalid (0.04)	Boscalid (0.03–0.03)
							Pyrimethanil (1.46–1.93)
							Imidacloprid (0.04)
6	26 July 2021	Royal Gala	1/5 (*n* = 2)	*K. ohmeri*	Triazoles	^*c*^	^*c*^
					Diazoles	^*c*^	^*c*^
					Other fungicides	Captan (0.12)	Captan (0.04–0.08)
7	26 July 2021	Red Delicious	1/5 (*n* = 1)		Triazoles	Sulfentrazone (0.03)	Sulfentrazone (0.01–0.02)
					Diazoles	Fludioxonil (0.02)	Thiabendazole (0.02–0.04)
						Thiabendazole (0.03)	Pyraclostrobin (0.01–0.02)
						Pyraclostrobin (0.04)	
					Other fungicides	Diphenylamine (0.36)	Diphenylamine (0.26–0.52)
						Pyrimethanil (0.93)	Pyrimethanil (0.55–1.16)
8	26 July 2021	Red Delicious	0/5		No detection	No detection	No detection
9	3 September 2021	Red Delicious	0/5		Triazoles	^*c*^	Tebuconazole^*b*^ (0.01–0.03)
					Diazoles	^*c*^	^*c*^
					Other fungicides	^*c*^	Biphenyl (0.01)
						^*c*^	Hexythiazox (0.01)
10	3 September 2021	Red Delicious	0/5		Triazoles	^*c*^	Tebuconazole^*b*^ (0.05)
						^*c*^	Difenoconazole^*b*^ (0.03)
					Diazoles	^*c*^	^*c*^
					Other fungicides	^*c*^	^*c*^
11	7 September 2021	Red Delicious	0/5		No detection	No detection	No detection
12	21 September 2021	Red Delicious	0/5		No detection	No detection	No detection

a Slot no. 1 to 8 represent apples purchased from local vendors (nonseasonal stored apples), slot no. 9 to 12 represent seasonal apples which were hand-picked from three apple orchards from apple-growing belts of Northern India, slot no. 9 to 10, from orchards used nonorganic farming, and slot no. 11 to 12 used organic farming.

b Demethylation inhibitor (DMI) triazoles.

c Negative for specific category of fungicide.

### Growth characteristics of *C. auris* strains on the surfaces of apples (CasSA).

All 16 CasSA showed oval yeast cells without pseudohyphae, similar to that of the C. auris reference strain B8441. The growth curve analysis of CasSA showed similar growth patterns, reaching stationary phase within approximately 20 h. Furthermore, all CasSA grew in the presence of 640 mg/L calcofluor white (CFW) and at 10% sodium chloride concentration.

**CasSA exhibit haploid genome.** Flow cytometry analysis of two randomly selected CasSA and B8441 showed geometric mean value of growth phase 1 (G_1_) cells ranging from 39,563 to 45,000 AU (arbitrary units), close to the geometric mean (40,794 AU) of the haploid C. glabrata (ATCC 15545) (see supplemental material, Fig. S1A to C). This result indicates that our CasSA are haploid.

10.1128/mbio.00518-22.5 FIG S1 Histograms representing DNA content obtained by FACS. Panels B1 and 2 show haploid C. auris reference clade I strains (B11098, B8441). Panels C1 and 2 show haploid C. auris strains on the surfaces of apples (CasSA). Panel A shows a haploid C. glabrata strain (ATCC 15545) for comparison, which was also analyzed. The *x* axis represents nuclear fluorescence, and the *y* axis represents cell number. Download FIG S1, DOCX file, 0.2 MB. Copyright © 2022 Yadav et al. 2022 Yadav et al. https://creativecommons.org/licenses/by/4.0/ This content is distributed under the terms of the Creative Commons Attribution 4.0 International license.

### Presence of fungicides, including triazole DMIs, in apples.

The results of gas chromatography-mass spectrometry (GC-MS) and liquid chromatography with tandem mass spectrometry (LC-MS/MS) showed a diversity of fungicides in the analyzed apples (Table 2). Specifically, of the 48 screened apples, 50% showed presence of triazole fungicides, including three 14α-demethylase inhibitors (DMIs), i.e., tebuconazole (TEB), difenoconazole (DEF), and flusilazole (FLU). Furthermore, we also detected different groups of diazole fungicides, such as methyl benzimidazole carbamates (carbendazim and thiabendazole), quinone outside inhibitors (pyraclostrobin), and fludioxonil, a phenolpyrrole. The fungicides were similarly distributed between apples with and without C. auris isolation. As expected, no fungicide was detected in freshly picked apples from organically farmed orchards, whereas triazole DMIs (TEB and DEF) were detected in the freshly picked apples from nonorganic orchards.

### CasSA show cross-resistance to agriculture triazole fungicides.

**FIG 1:**
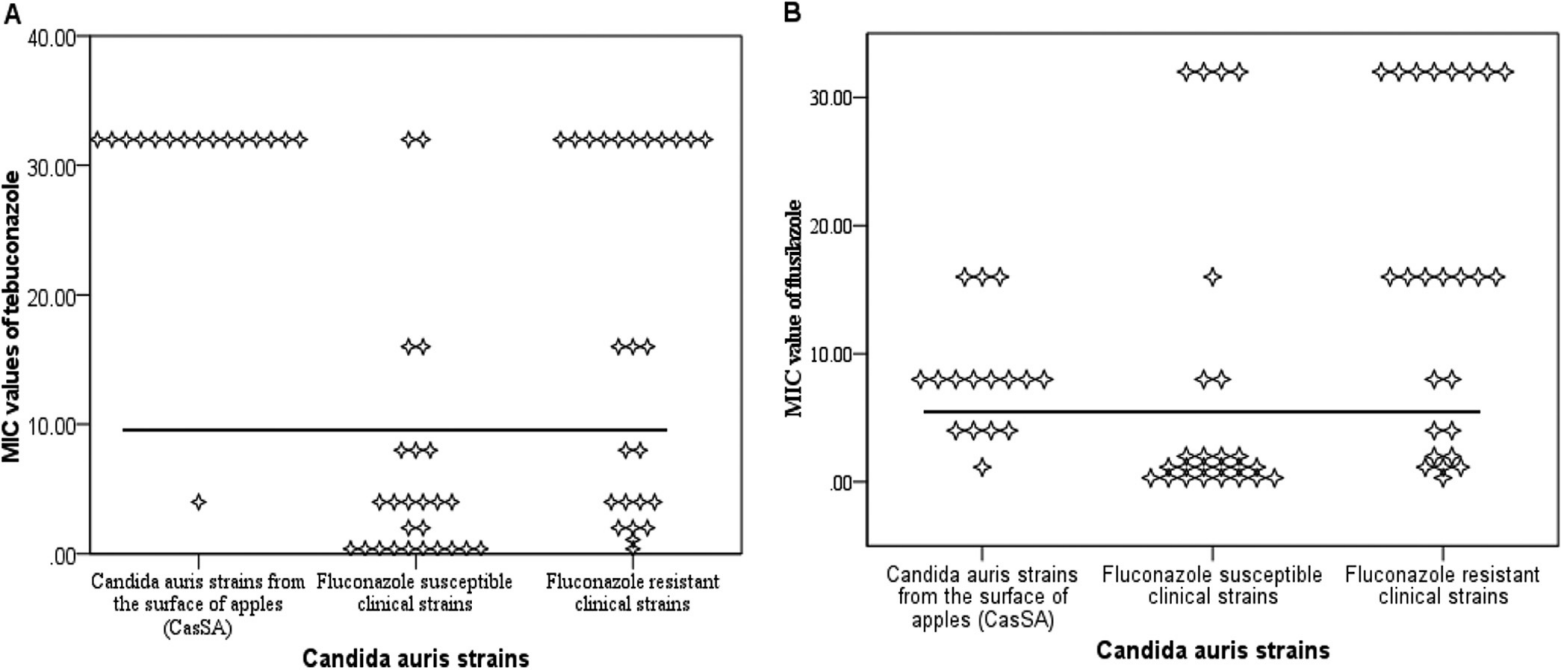
Scatterplot depicting MIC (mg/L) distribution of two DMIs, (A) tebuconazole and (B) flusilazole, against C. auris strains from surfaces of apples (CasSA, *n* = 16), 25 fluconazole-susceptible clinical strains (MIC, <16 mg/L) and 25 fluconazole-resistant clinical strains (MIC >32 mg/L).

We compared the antifungal susceptibilities of medical antifungals and agriculture triazole fungicides for the 16 CasSA with clinical and marine environmental strains from India (7, 14). Except one isolate that had a fluconazole (FLC) MIC of 16 mg/L, the remaining 15 CasSA all had high FLC MICs, >128 mg/L. Further, high MICs of voriconazole (VRC; MIC of ≥2 mg/L) and amphotericin B (AMB; MIC ≥2 mg/L) were observed in 50% and 44% of the 16 CasSA, respectively (Table 3). Antifungal susceptibility profiles of other yeast species isolated from fruits are given in Table 4. For agriculture triazole fungicides, the 16 CasSA also showed an overall low *in vitro* susceptibility. Specifically, high geometric mean (GM) MIC values were observed for the three triazole fungicides (DMIs): tebuconazole (TEB; GM MIC of 45.25 mg/L), bromuconazole (BRO; GM MIC of 13.45 mg/L), and flusilazole (FLU; GM MIC 6.72 mg/L). Interestingly, among these three DMIs, two (TEB and FLU) were detected in the apples analyzed in the present study. Similarly, the 16 CasSA had a high GM MIC value of 128 mg/L against the diazole fungicide carbendazim, also detected in our analyzed apples. Cross-resistance was also observed for the clinical and environmental strains used here for comparison. For example, the 25 clinical C. auris strains resistant to FLC (MIC of >32 mg/L) all exhibited high MICs against agriculture DMIs, with high GM MIC values of 11.47 mg/L for TEB, 8.94 mg/L for FLU, and 3.29 mg/L for BRO (Table 3 and Fig. 1). Interestingly, statistically significant (*P* = 0.0001) 4-fold-higher GM MIC values of TEB and BRO were observed in the CasSA population compared to those in 25 FLC-resistant clinical C. auris strains (Table 3 and Fig. 1). Similarly, C. auris strains from natural marine environment distant from agriculture with no fungicide usage had statistically significant lower (2- to 9-fold; *P* = 0.0001) GM MICs of triazole fungicides than the CasSA population.

**TABLE 3:** MIC distribution of medical antifungals and agriculture azoles against 16 C. auris strains from surfaces of apples, clinical C. auris strains (*n* = 50), and strains from marine environment (*n* = 10, Andaman Islands, India)

C. auris (no. of isolates)	Parameter	MIC distribution of medical antifungal (mg/L)^*a*^							MIC distribution of agriculture azole (mg/L)^*b*^						
									DMI triazoles					Diazoles	
		FLC	VRC	ITC	ISA	POS	AMB	5-FC	TEB	BRO	EPX	PCZ	FLU	PCL	CBZ
C. auris strains from surface of apples (*n* = 16)	Range	16–128	1–16	1–32	0.25–4	0.25–2	0.25	0.12–0.25	4–64	1–32	0.25–4	0.25–2	01–16	0.25	128
	^*c*^GM	112.40	1.04	0.88	0.23	0.08	1.35	0.14	45.25	13.45	1.83	1.19	6.72	0.25	128
	^*d*^MIC_50_	128	8	16	2	2	0.25	0.12	64	16	2	2	8	0.25	128
	^*e*^MIC_90_	128	16	32	4	2	0.25	0.18	64	32	4	2	16	0.25	128
Marine environmental strains (*n* = 10)	Range	128	0.5–4	01–4	0.03–0.50	0.03–0.25	0.25–4	0.03–64	16–128	1–32	0.25–16	0.25–4	2–32	0.25	128
	GM	105.95	1.20	0.88	0.36	0.22	3.75	28.21	19.32	1.45	0.36	0.32	2.57	0.25	128
	MIC_50_	128	2	1	0.50	0.25	4	16.03	64	2	1.50	1	2	0.25	128
	MIC_90_	128	4	4	0.50	0.25	4	64	83.20	15.20	6.2	2.6	20.8	0.25	128
FLC-resistant clinical strains (*n* = 25; MIC, >32 mg/L)	Range	32–128	0.06–2	0.03–1	0.01–0.50	0.01–0.25	0.12–8	0.12–64	0.25–64	0.25–16	0.25–8	0.25–8	0.12–64	0.25	128
	GM	84.44	0.47	0.15	0.07	0.06	0.57	1.43	11.47	3.29	0.82	0.87	8.94	0.25	128
	MIC_50_	128	0.50	0.12	0.06	0.06	0.50	0.50	16	4	1	1	16	0.25	128
	MIC_90_	128	2	0.5	0.40	0.25	1	64	64	12.80	3.20	3.20	32	0.25	128
FLC-susceptible clinical strains (*n* = 25; MIC, <16 mg/L)	Range	0.25–16	0.03–1	0.03–1	0.01–0.25	0.01–0.5	0.25–4	0.12–64	0.25–32	0.25–8	0.25–2	0.25–4	0.12–64	0.25	128
	GM	2	0.08	0.12	0.03	0.05	0.87	0.34	1.89	0.77	0.34	0.37	1.84	0.25	128
	MIC_50_	2	0.03	0.06	0.03	0.03	0.50	0.12	4	1	0.25	0.25	1	0.25	128
	MIC_90_	12.8	0.50	0.50	0.25	0.25	2	6.40	16	6.40	0.80	1.60	32	0.25	128

a FLC, fluconazole; VRC, voriconazole; ITC, itraconazole; ISA, isavuconazole; POS, posaconazole; AMB, amphotericin B; 5FC, flucytosine.

b TEB, tebuconazole; BRO, bromuconazole; EPX, epoxiconazole; PCZ, propiconazole; FLU, flusilazole; PCL, pyraclostrobin; CBZ, carbendazim. TEB to carbendazim.

c Geometric mean MICs.

d MIC_50_, MIC at which 50% of tested isolates were inhibited.

e MIC_90_, MIC at which 90% of tested isolates were inhibited.

**TABLE 4:** *In vitro* antifungal susceptibility profile of 12 yeast species (*n* = 112) isolated from nine fruit plant species against 10 antifungal drugs using CLSI-BMD method

Slot no.	Species (no. of strains tested)	Parameter	MIC (mg/L)^*a*^									
			FLC	VRC	ITC	ISA	POSA	AFG	MFG	CFG	AMB	5-FC
1	C. guilliermondii (*n* = 27)	Range	0.25–64	0.03–0.12	0.03–0.50	0.01–0.25	0.01–0.25	0.25–4	<0.01–1	0.50–2	0.06–0.50	<0.03–2
		^*b*^GM	3.61	0.05	0.33	0.09	0.13	1.32	0.43	1.39	0.36	0.21
		^*c*^MIC_50_	2	0.06	0.50	0.12	0.25	2	0.50	2	0.50	0.12
		^*d*^MIC_90_	16	0.12	0.50	0.25	0.25	4	1	2	0.50	1.20
2	C. parapsilosis (*n* = 23)	Range	0.25–2	0.06–0.12	0.03–0.12	0.01	0.01–0.25	0.06–4	0.03–2	0.50–4	0.03–0.50	0.03–0.12
		GM	0.61	0.08	0.07	0.01	0.02	0.48	0.44	1.57	0.13	0.04
		MIC_50_	0.50	0.09	0.06	0.01	0.01	0.50	0.50	2	0.25	0.03
		MIC_90_	2	0.12	0.12	0.01	0.02	1	1	2	0.25	0.12
3	C. tropicalis (*n* = 19)	Range	0.25–2	0.03–0.06	0.03–0.25	0.01–0.25	0.01–0.25	0.03–0.25	0.01–0.03	0.50–2	0.06–0.50	0.03–0.25
		GM	0.59	0.04	0.09	0.05	0.05	0.07	0.02	1.2	0.16	0.11
		MIC_50_	0.50	0.03	0.12	0.03	0.06	0.06	0.01	1	0.25	0.12
		MIC_90_	2	0.06	0.25	0.25	0.25	0.12	0.02	2	0.25	0.25
4	*Kodamea ohmeri* (*n* = 14)	Range	1–8	0.03	0.03–0.12	0.01–0.03	0.01–0.03	0.03–0.50	0.01–2	1–4	0.03–0.12	0.03–0.12
		GM	2	0.03	0.12	0.02	0.01	0.13	0.06	1.70	0.06	0.12
		MIC_50_	2	0.03	0.12	0.02	0.01	0.12	0.06	1	0.06	0.12
		MIC_90_	2	0.03	0.12	0.03	0.02	0.42	0.12	4	0.12	0.12
5	C. lusitaniae (*n* = 7)	Range	<0.25–8	0.03	<0.03–0.06	<0.01–0.03	<0.01–0.03	0.50–1	0.50–1	0.12–0.50	0.50–1	0.12
6	*Lodderomyces elongisporus* (*n* = 4)	Range	2–4	0.03–0.12	0.25	>0.01	0.06–0.50	0.01	0.01	0.50	0.12	0.12
7	C. carribica (*n* = 4)	Range	2–8	0.06–0.12	0.25–1	0.03–0.50	0.06–0.50	0.25–2	0.25–0.50	2	0.50	0.03
8	*C. carpophila* (*n* = 3)	Range	8	0.06	0.25	0.12	0.06	0.50	0.25	2	0.50	0.03–0.06
9	*Merozyma ferinosa* (*n* = 3)	Range	0.25–8	0.03	0.03	<0.01–0.03	0.01	0.50	0.25–0.50	0.12–0.50	1	<0.12
10	*C. rugosa* (*n* = 2)	Range	1	<0.03	<0.03	<0.01	0.01	0.12	0.03	1	0.25	0.50
11	C. kefyr (*n* = 2)	Range	0.12	<0.03	<0.03	<0.01	0.03	0.25–0.50	0.06	1	0.25–0.50	0.25–4
12	*C. blankii* (*n* = 1)	MIC	8	0.06	0.12	<0.01	0.06	2	2	1	0.50	0.12
13	C. krusei (*n* = 1)	MIC	32	0.12	0.50	<0.01	0.12	0.12	0.25	2	1	8
14	C. albicans (*n* = 1)	MIC	4	0.25	0.06	0.01	0.01	1	0.12	1	0.25	0.06
15	Trichosporon ashaii (*n* = 1)	MIC	0.25	<0.03	0.06	0.01	0.25	2	2	0.50	0.50	0.12

a FLC, fluconazole; VRC, voriconazole; ITC, itraconazole; ISA, isavuconazole; POS, posaconazole; AFG, anidulafungin; MFG, micafungin; CAS, caspofungin; AMB, amphotericin B; 5FC, flucytosine.

b Geometric mean MICs.

c MIC_50_, MIC at which 50% of tested isolates were inhibited.

d MIC_90_, MIC at which 90% of tested isolates were inhibited.

A remarkable pattern noted was that clinical C. auris strains that were susceptible to FLC (GM MICs of 2 mg/L) also showed low GM MICs (0.34 to 1.89 mg/L) for all agriculture triazole fungicides tested. In fact, 3-fold and 24-fold decreases in GM MICs of TEB and BRO, respectively, were noticed for clinical strains susceptible to FLC compared to those of CasSA (*P* ≤ 0.0001). Interestingly, a single strain (VPCI/F1/B/2020) of C. auris recovered from apple which was less resistant to FLC (MIC 16 mg/L) also showed low MIC values for all triazole fungicides tested, i.e., BRO (1 mg/L), FLU (1 mg/L), and TEB (4 mg/L).

### Genomic analyses of C. auris.

The whole-genome sequences of the 16 CasSA isolated in this study as well as those of 43 previously reported Indian C. auris strains were analyzed and compared using the NASP pipeline (15). Candida auris strain B8441 assembly V2 was used as the reference clade I strain. The 43 previously published Indian strains included 25 from patients, 5 from hospital environments, and 13 from the natural marine environment from Andaman Island (2, 4, 7).

**FIG 2:**
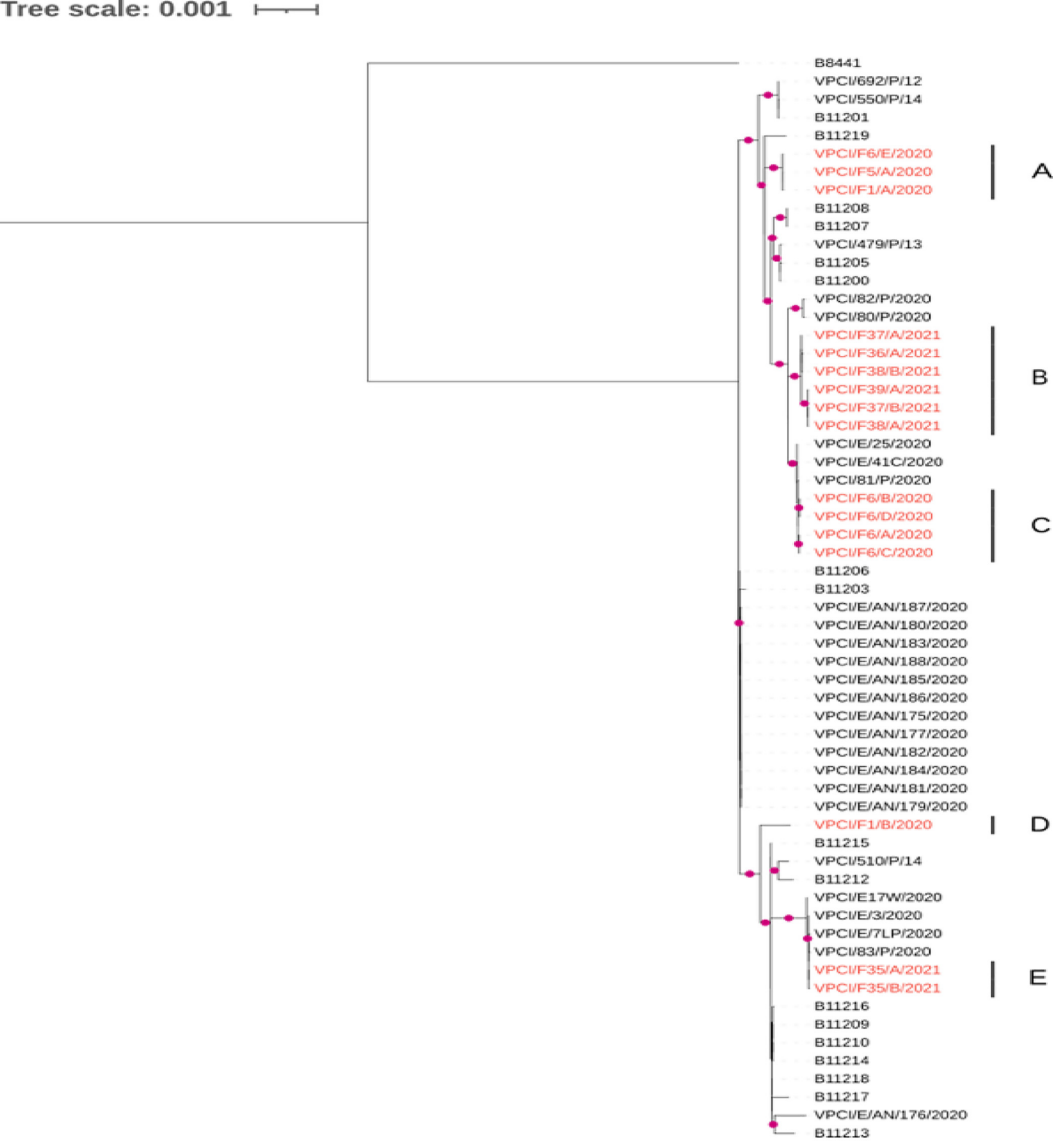
Maximum-likelihood phylogenetic tree of 60 C. auris strains was constructed by using RAxML v8.0.25. Included in the tree are 16 C. auris strains from surfaces of apples (CasSA), 13 environmental strains from Andaman Islands, India, and 30 Indian clinical strains along with reference strain, B8441. The tree was constructed based on the 1,281 shared SNPs among the 59 strains. Branches with bootstrap support over 75% of 100 bootstrap iterations are labeled with red markers. CasSA were highlighted in red and clustered in 5 subclusters (A to E).

**FIG 3:**
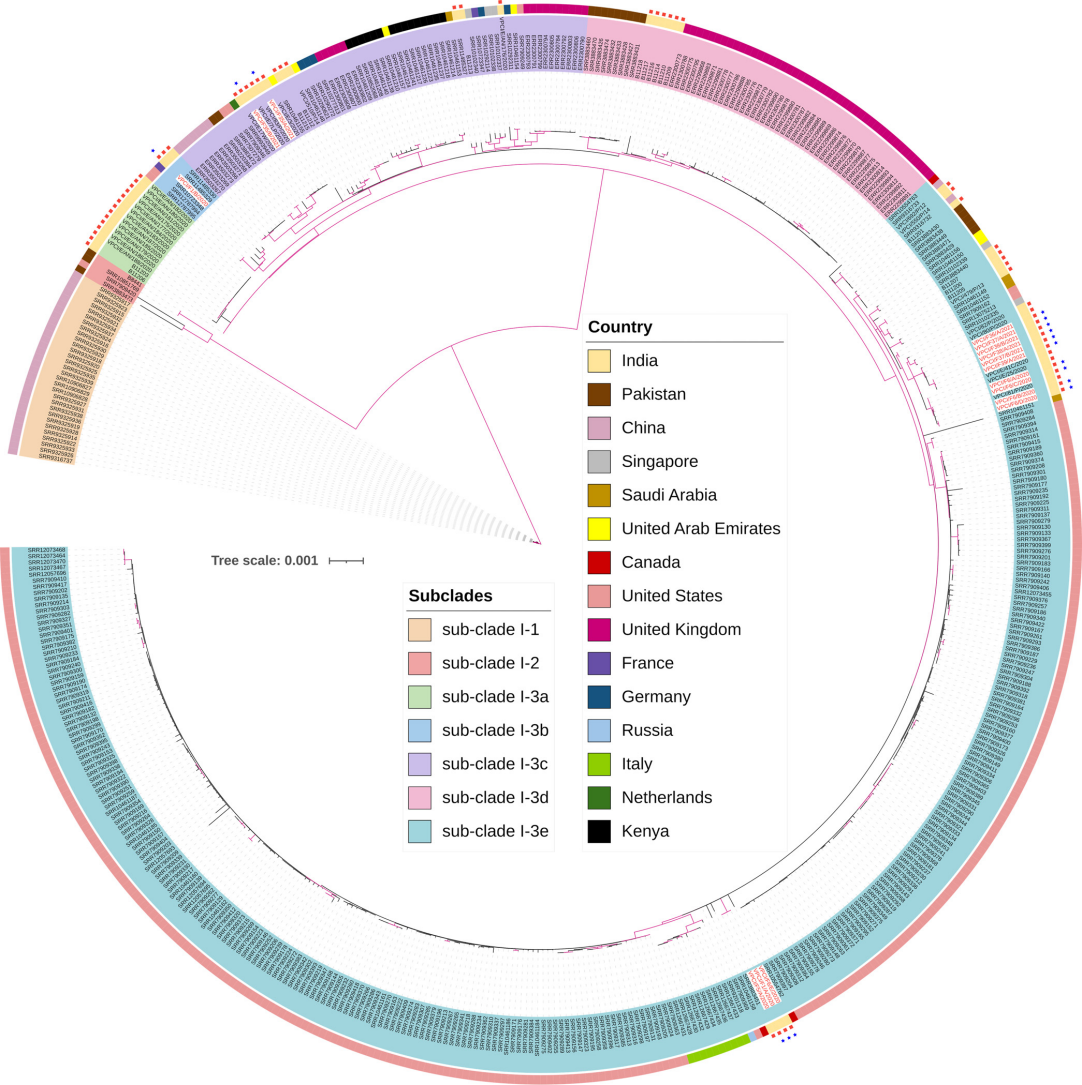
Maximum-likelihood phylogenetic tree showing the relationships among 503 clade I Candida auris isolates from around the globe. The isolates’ relationships were inferred based on their whole-genome single nucleotide polymorphisms. Here, based on their branch lengths and bootstrap support values, the 503 isolates were further classified into 7 subclades, including three major subclades (subclade I-1, subclade I-2, and subclade I-3), with subclade I-3 containing five more recently derived ones (subclades I-3a, I-3b, I-3c, I-3d, and I-3e). Isolates within each subclade are highlighted with the same background color over the isolate identifications. The color strip outside the isolate identification indicates the country of origin for each isolate. In addition, isolates from India are highlighted with red squares. Furthermore, the isolates from apples in India are marked with blue stars, placed adjacent to their red square labels. Branch lengths are proportional to the number of SNP differences among strains. Branches in magenta have a bootstrap support above 75%.

**Genetically distinct CasSA belonged to different subclades within clade I.** The maximum-likelihood phylogeny clustered all 16 CasSA as belonging to clade I, consistent with the grouping of other C. auris strains from India. The 16 CasSA formed five distinct genotype clusters (A to E), with four clusters consisting of more than one CasSA isolate each; cluster D was the exception, which contained a single CasSA, VPCI/F1/B/2020. Out of the 16 CasSA strains, 10 from seven apples (F1, F5, F6, F36, F37, F38, and F39) belonged to three clusters (A, B, and D) with no known closely related genotypes from other sources in India (Fig. 2). These apple-specific genotype clusters differed by 28 to 157 single nucleotide polymorphisms (SNPs) with the previously reported clinical, hospital inanimate environment, and natural marine environment strains from India, indicating apple surfaces harboring unique C. auris strains. In a broad analysis including 487 global clade I C. auris strains with genome sequences deposited in GenBank as of August 2021, we separated these strains into seven subclades each with at least 75% bootstrap support values. The details of 487 global clade I C. auris are given in the supplemental material, Table S1. The 16 CasSA clustered into three of the seven subclades, and their closest genotypes were all Indian clade I strains, with genome-wide SNP differences ranging from 0 to 157 between individual CasSA and their closest genotype from India (Fig. 3).

10.1128/mbio.00518-22.1 TABLE S1 Details of the 503 global isolates of C. auris (specimens, country of origin, and bio-project numbers) included for genomic analysis in the present study. Download Table S1, XLSX file, 0.04 MB. Copyright © 2022 Yadav et al. 2022 Yadav et al. https://creativecommons.org/licenses/by/4.0/ This content is distributed under the terms of the Creative Commons Attribution 4.0 International license.

**Genetically similar C. auris strains on different varieties of apples procured from various sources.** Genetically identical strains (0 SNP) were observed on the surfaces of two groups of three apples each. One group included strains from apples F1, F5, and F6 (genotype cluster A). These three apples contained both varieties of apples tested, i.e., Red Delicious and Royal Gala, and they were procured from two different vendors separated by 30 km. The second group of genetically identical strains included those from apples F37, F38, and F39 (genotype cluster B). In addition, genotype cluster B also included a genetically highly similar strain from apple F36 (2 to 9 SNPs), and together these four apples (F36 to 39) including both apple varieties were procured from three geographically separated vendors. We note that Delhi and NCR vendors purchase fruits from the common National Fruit and Vegetable Market, one of the biggest wholesale fruit and vegetable markets in Asia, located in North Delhi.

**Heterogenous population of clade I C. auris strains on the surface of individual apples.** Interestingly, while several C. auris strains from different apples shared identical genotypes, we also observed that several apples contained strains of C. auris with different genotypes. Specifically, apple F6 harbored three genotypes belonging to two clusters which differed by as many as 59 SNPs. Similarly, the two strains from apple F1 differed by 107 SNPs. For the three apples (F35, F37, and F38) where two colonies each of C. auris were isolated, the pairs of strains differed by 1, 7, and 8 SNPs, respectively, for those from apples F35, F37, and F38. Together, the results here are consistent with the colonization of apples by genetically distinct strains as well as microevolution of the strains after their colonization on individual apples.

**Candida auris strains on surfaces of apples are related to clinical strains.** Out of 16 CasSA, 6 from two apples (F6 and F35) fell into two genotype clusters, C and E, which included both clinical and fruit strains. In cluster C, four clonal CasSA (0 to 3 SNP difference) recovered from a single apple clustered with three clonal C. auris strains recovered from a single hospitalized patient (VPCI/81/P/2020) and his inanimate hospital environment (0 to 3 SNP differences). Interestingly, both clinical and CasSA strains in cluster C were identical, with 0 to 3 SNP differences, consistent with their recent shared ancestry. Similarly, in genotype cluster E, two clonal CasSA (1 SNP difference) recovered from the surface of apple F35 were found to be highly related (0 to 3 SNP differences) with four clonal C. auris strains recovered from the ear of a patient (VPCI/83/P/2020) and his immediate inanimate environment. Together, these observations are consistent with the stored apples serving as a possible reservoir for transmission of C. auris strains among people and among ecological niches.

While genetically the closest clinical strains to the 16 CasSA strains were those reported from India, several strains from outside India also showed high similarities to some of the CasSA strains. For example, one strain from France was highly similar to fruit strain VPCI/F1/B/2020. Similarly, two strains from Canada showed high SNP similarities to genetic cluster A of the CasSA, comparable to the closest strain from India (Fig. 3).

**FIG 4:**
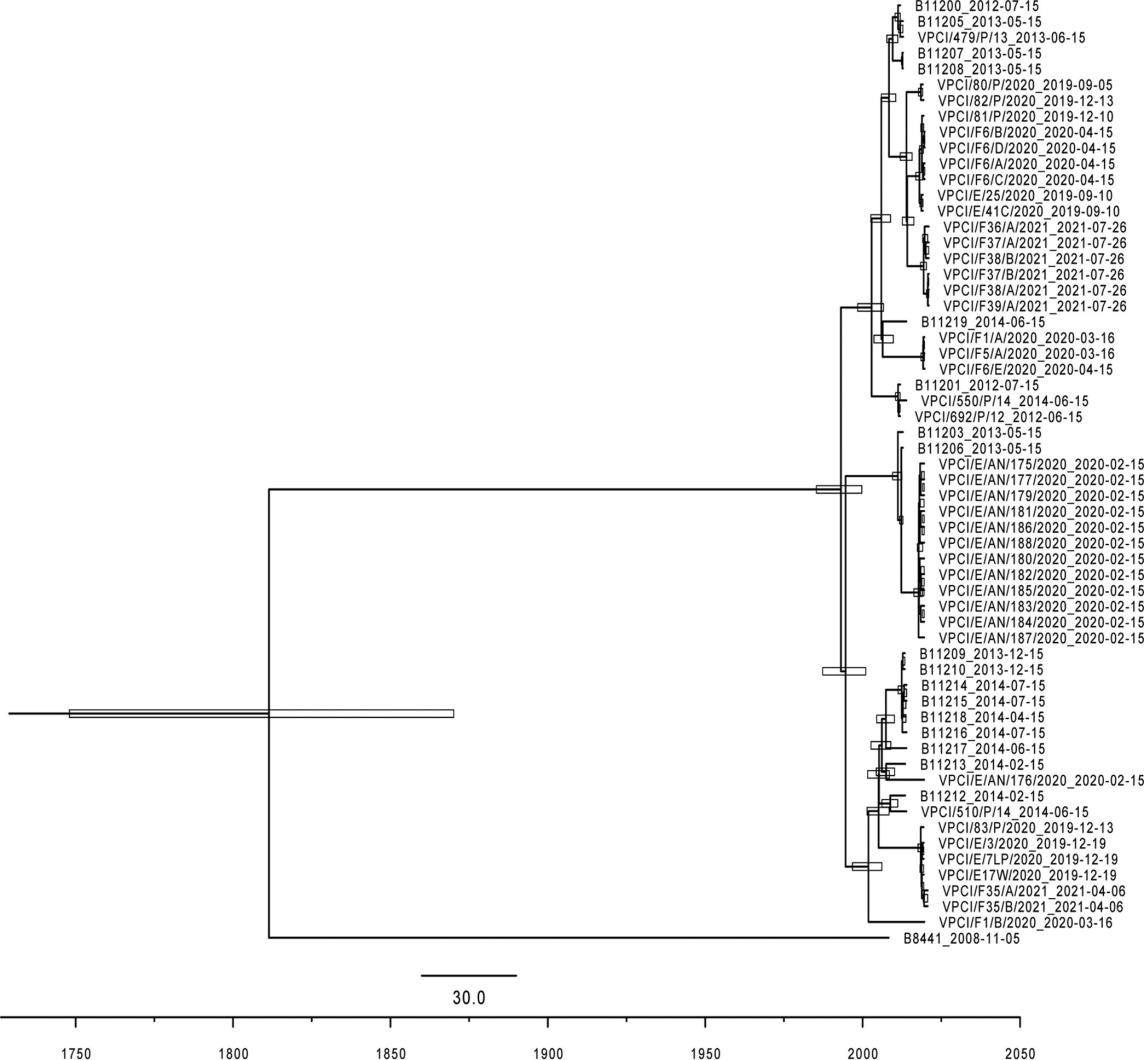
Maximum clade credibility phylogenetic tree of 16 CasSA isolated in the present study, and 43 previously published Indian C. auris strains from both clinical and natural marine environments along with clade I reference strain B8441.

**Divergence time estimation of fruit isolates showed their emergence in the last 2 decades.** The divergence time of the 16 CasSA from the other 43 Indian strains was inferred using evolutionary model and Markov Chain Monte Carlo (MCMC) analysis in BEAST v2.6.3 based on the concatenated sequences of only genes containing SNPs (16). The specimen collection dates were used as the sampling dates and a strict molecular clock model, and an exponential prior distribution on the clock rate was applied. In addition, we adopted a general time reversible nucleotide substitution and coalescent exponential population model. The emerging dates of the fruit isolates were estimated by calculating time to the most recent common ancestor (TMRCA) of other Indian isolates. All 16 CasSA were grouped into five clusters (Fig. 4). Cluster A, consisting of three C. auris strains from three different apples with sample B11219, formed an outgroup of the cladogram including clusters B and C, and they diverged from the cladogram in 2005 (12.2 to 18.4, 95% highest posterior density [HPD]). Cluster B is the sister group to cluster C. They diverged from each other in 2014 (4.7 to 8.4 years ago, 95% HPD). Cluster A and B11219 diverged from each other in 2006 (11.3 to 17.4, 95% HPD). Cluster E, containing two CasSA and 4 Indian clinical samples, recently emerged and formed a bigger cladogram around 2018 (1.8 to 3.4, 95% HPD), which is the outgroup for other 10 Indian clinical samples and one environment sample from Andaman Islands. The estimate divergence time of the 11 samples and the outgroup is around 2005 (12.6 to 19.6, 95% HPD). Cluster D contained a single strain, VPCI/F1/B/2021, and diverged earlier than other fruit isolates, around 2002 (14.9 to 24.2, 95% HPD). The TMRCA frame of 2002 to 2005 overlaps the rise in health care antibiotic consumption in India starting around 2004 to 2006. (17).

**Genetic determinants of azole resistance in CasSA.** Interestingly, CasSA showed similar genetic determinants of antifungal resistance as previously reported in clinical C. auris strains. CasSA strains showed amino acid substitutions K143R (*n* = 13) or Y132F (*n* = 3) in the azole target *ERG11* gene along with amino acid substitution V704L (*n* = 13) or E709D (*n* = 3) in *CDR1* gene (14). In the zinc-cluster transcription factor *TAC1B*, amino acid substitution A640V was observed (18). In addition, two CasSA (VPCI/F35/A/2021, VPCI/F35/B/2021) obtained from the same apple showed an amino acid substitution, G145D, in the *YMC1* gene. These two isolates clustered in the phylogenetic tree (Fig. 2) with clinical C. auris isolates that also had the same substitution (2). *YMCI* is associated with several transmembrane transporter activities and is essential in mitochondrial transport as well as important for glutamate metabolism in Baker’s yeast Saccharomyces cerevisiae (19).

### Cuticular wax components of C. auris-positive and -negative apples.

A total of five apples were investigated by GC-MS for their surface wax components, including two C. auris-positive apples and three C. auris-negative apples (two freshly picked apples from organic orchards and one stored apple). No specific wax component was present in C. auris-colonized versus non*-*C. auris-colonized apples. Further, natural or synthetic wax components could not be differentiated in stored versus freshly picked apples due to the presence of similar components in the natural and artificial wax. Among fatty acids, palmitic acid (C16:0) was detected in all five apples. Overall, a total of 41 aliphatic components were identified in all apples (see supplemental material, Table S2).

10.1128/mbio.00518-22.2 TABLE S2 Characterization of aliphatic components in five apples, including two C. auris-positive and three C. auris-negative apples representing stored (*n* = 3) and freshly picked (*n* = 2) from organic orchards. Download Table S2, DOCX file, 0.01 MB. Copyright © 2022 Yadav et al. 2022 Yadav et al. https://creativecommons.org/licenses/by/4.0/ This content is distributed under the terms of the Creative Commons Attribution 4.0 International license.

### Metabarcoding analyses of fungal communities on the surface of apples from nonorganic orchards.

As stated above, we were unable to obtain a single C. auris colony from 20 freshly picked apples. However, the lack of success in isolating C. auris could be due to the low population density and/or low viability of C. auris on the surfaces of those freshly picked apples. To investigate this possibility, we directly analyzed the fungal communities on the surfaces of two apples using the culture-independent metagenome barcoding approach based on the internal transcribed spacer 1 (ITS1) sequences (12). Overall, the results were consistent with the culture results in that no ITS1 sequence matched that of C. auris from either apple. In addition, similar to culture results, several *Candida* species were detected, including C. orthopsilosis, C. albicans, C. glabrata, and C. krusei (Pichia kudriavzevii). The detailed fungal species distributions obtained from these two apples are given in the supplemental material (Tables S3 and S4).

10.1128/mbio.00518-22.3 TABLE S3 Taxonomic classification of fungal species detected from the surface of freshly picked apple 1 from organic orchards. Download Table S3, DOCX file, 0.01 MB. Copyright © 2022 Yadav et al. 2022 Yadav et al. https://creativecommons.org/licenses/by/4.0/ This content is distributed under the terms of the Creative Commons Attribution 4.0 International license.

10.1128/mbio.00518-22.4 TABLE S4 Taxonomic classification of fungal species detected from the surface of freshly picked apple 2 from organic orchards. Download Table S4, DOCX file, 0.01 MB. Copyright © 2022 Yadav et al. 2022 Yadav et al. https://creativecommons.org/licenses/by/4.0/ This content is distributed under the terms of the Creative Commons Attribution 4.0 International license.

## DISCUSSION

Our results demonstrate that the surfaces of stored apples represent a novel reservoir of transmission of C. auris and show unequivocally that some of the strains from stored apples belong to the same clonal groups as clinical isolates from India. In addition, high levels of antifungal resistance are common among these environmental isolates from apples. The results expand our understanding of the ecology of C. auris and should help develop a better strategy to minimize the spread of this multidrug-resistant fungal pathogen. The successful isolation of C. auris from apple surfaces is not surprising. Ecologically, yeasts are broadly distributed. Candida auris belongs to the *Clavispora* clade of the Metschnikowiaceae family, a group of yeasts isolated principally from nonhuman sources such as plants (both living and dead) and marine environments (7, 20). Previous studies have shown that several *Metschnikowia* spp., including Metschnikowia pulcherrima, Metschnikowia sinensis, and Metschnikowia fructicola, and Candida pruni of the Clavispora clade are commonly associated with fruits (21, 22). For example, both M. pulcherrima and M. sinensis have been isolated from apples (23). Interestingly, most of these isolations have come from Southeast Asia, in the tropical environments. Specifically, together with results from our previous report on the isolation of C. auris from the salt marsh with extensive vegetation, plants in tropical marine wetlands could represent a significant source of C. auris as originally proposed by Casadevall et al. (8, 24).

**FIG 5:**
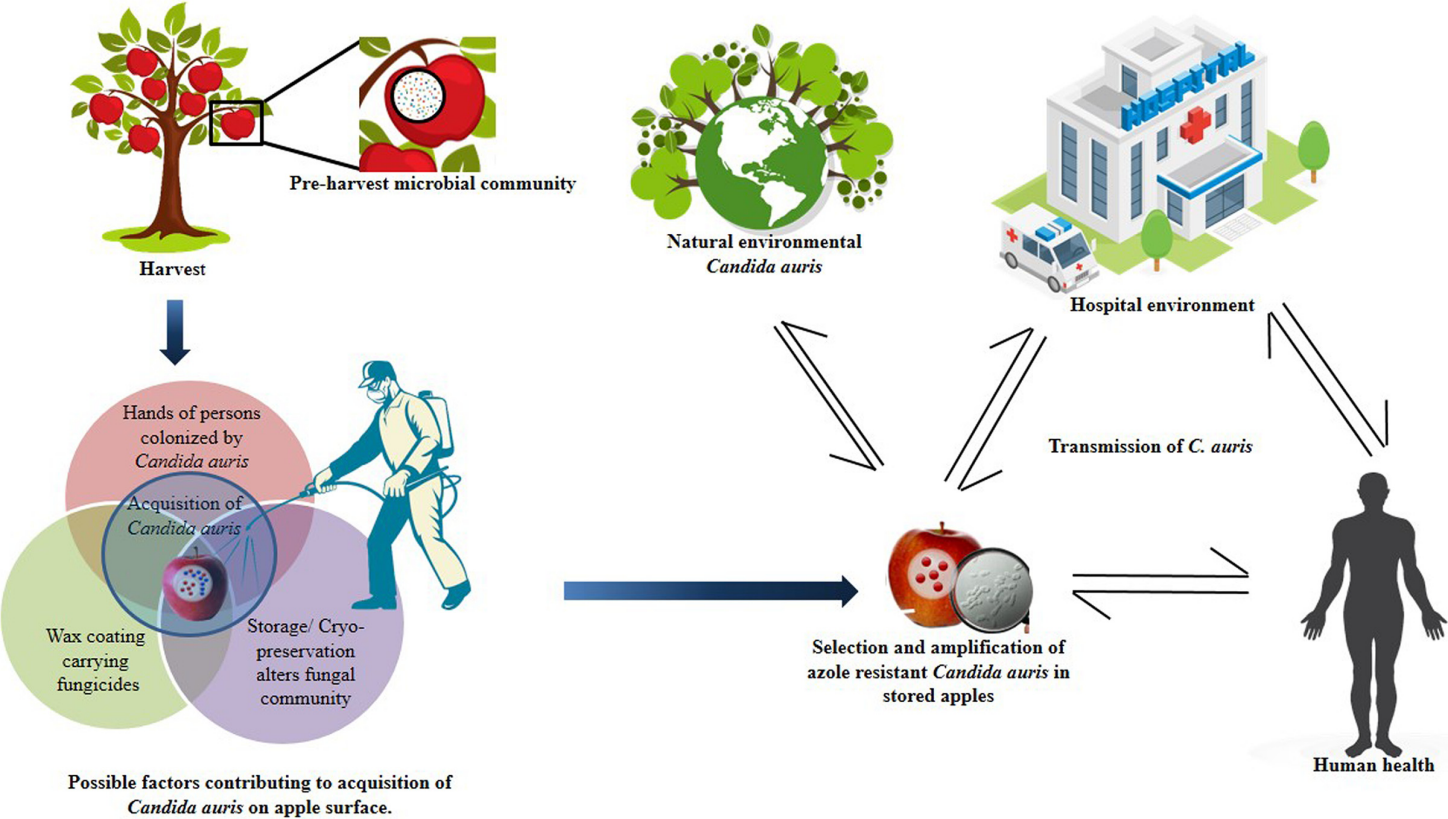
Schematic representation of stored apples as a possible reservoir of selection and transmission of azole-resistant C. auris.

The present study further documents the occurrence of C. auris isolates with reduced sensitivity to major DMI fungicides on the surface of apples. As expected, a broad range of fungicides were detected in the screened apples, including three triazole DMIs, namely, tebuconazole, difenoconazole, and flusilazole. DMIs share molecular structure characteristics to medical triazoles, and in the agriculture settings, these fungicides have been proposed as a selective force for cross-resistance to medical azoles in human-pathogenic fungi such as Aspergillus fumigatus (25). Indeed, previous studies have shown that cross-resistance to medical triazoles can also be achieved *in vitro* by exposing yeasts to agriculture azoles. For example, a recent report demonstrated that strains of the C. parapsilosis species complex exposed to DMIs (tebuconazole and tetraconazole) for 15 days developed reduced fluconazole susceptibility (26). Similarly, exposure of C. parapsilosis to tetraconazole, an agriculture triazole, led to decreased susceptibility to three medical triazoles, i.e., fluconazole, itraconazole, and voriconazole (27). Candida auris strains in the present study exhibited fluconazole resistance as well as high MICs toward voriconazole. We believe that the azole fungicides on the surface of apples have likely contributed to the observed high triazole MICs in these C. auris strains. Indeed, large amounts of fungicides are used worldwide in apple cultivation to control fungal diseases on apples, such as apple scab caused by filamentous fungus Venturia inaequalis. A recent study reported that a long-term fungicide use led to decreased sensitivity of V. inaequalis strains to multiple fungicides, including DMIs, in field conditions (28). In the present study, all *C. auris* strains excepting one from apples showed resistance to three triazole fungicides (DMIs), i.e., tebuconazole (GM MIC of 45.25 mg/L), bromuconazole (GM MIC of 13.45 mg/L), and flusilazole (GM MIC of 6.72 mg/L). Our findings suggest that C. auris in the natural ecosystem may come in contact with agriculture fungicides and fruits may be a potentially significant niche for the development of azole resistance in C. auris (Fig. 5).

This study observed that 13% (*n* = 8/62) of the apples were positive for C. auris, yielding a total of 16 C. auris strains from surfaces of eight apples. Genomic analyses revealed both genetically distinct strains and closely related clonal clusters, with some of the clonal genotype clusters consisting of strains from different apples, including apples of different varieties and/or from different vendors. The results suggest that either the C. auris strains were from the common wholesale fruit market in Delhi and NCR or that long-distance strain dispersal is common for this species in this geographic region. Interestingly, C. auris was recovered only from stored apples, and none of the freshly harvested apples collected from conventional and organic farms was positive for C. auris. In fact, the metabarcoding (microbiome) analysis of fresh apples showed negative results for C. auris. Thus, the most likely hypothesis about the origin of C. auris on the surface of stored apples is the contamination of apples by fingers and palms of humans who were colonized with C. auris during the postharvest treatment and storage and through the distribution chain. However, the genetic uniqueness of many of the strains suggests that there were likely multiple sources of C. auris contamination on the stored apples. Further, it is important to emphasize that in the stored apples the fungal diversity is likely prone to change over time (29). Indeed, the composition of the postharvest microbiome of apples is known to be affected by bacterial and fungal communities present in packing houses where fruits are processed for market and storage after harvest (29). For example, a high prevalence of medically important yeast genera (*Malassezia*, *Candida*, and *Trichosporon*) associated with human infections was observed on Red Delicious apples sampled from a local supermarket in West Virginia, United States, but the source of these genera from handling prior to or after arriving at the supermarket could not be determined (29). It should be noted that only a small number of freshly harvested apples were screened in the present study and our understanding of the natural microbiota of our apple cultivars in India is very limited. As a result, it is difficult to interpret if the surface of stored apples represents a significant reservoir of C. auris. In addition, we were unable to identify the source of C. auris from handling prior to our purchase from the local vendors. It is plausible that multiple practices, like cryopreservation and wax coating with additional fungicides during the storage of apples, may alter the myco-flora of apples’ surfaces. Subsequently, C. auris could be amplified under the directional selection pressure of fungicides. Interestingly, in four of the eight apples, C. auris was the only observed yeast, suggesting a potential competitive dominance of C. auris over other yeasts on the surfaces of apples. Indeed, on human skin, commensal *Malassezia*-dominated communities were replaced by communities dominated by C. auris soon after the invasion by C. auris, including a significant reduction of other *Candida* species (30).

Common practices used to maintain quality and increase shelf life of stored apples include precooling, washing, sanitizing, and waxing. With these treatments, apples can be stored for 6 to 12 months at low temperatures (1 to 2°C) before distribution. These procedures can affect the natural protective wax layer that covers a fruit’s surface (31). Therefore, it is a common practice to apply a thin layer of edible wax, consisting of esters of a higher fatty acid with monohydric alcohols, hydrocarbons, and free fatty acids, to serve the same function as natural wax, i.e., covering fruit injuries, reducing water loss, and adding a shine/gloss to the fruit surface (31). Interestingly, East Asian and Iranian clades of C. auris are found almost exclusively in the internal ear canal, which also contains natural ear wax (19). Candida auris association with natural wax components may explain the presence of this yeast in waxed/stored apples. In fact, linoleic acid, oleic acid, palmitic acid, and stearic acid are the major free fatty acids which are common in both ear wax and apple cuticular wax (32, 33).

In addition, wax has been used as a carrier of fungicides and no significant change in the amount of fungicide residues was observed during a 40-day storage of waxed apples under ambient conditions (13). In the present study, in stored apples 12 different fungicides were detected, whereas freshly picked nonorganic apples had 4 types of fungicides. The additional classes of fungicides on stored apples were likely added during postharvest treatments of the stored apples. These additional fungicides on stored apples could act as strong selective forces for multidrug-resistant species such as C. auris.

CasSA strains showed substantial genetic diversity and broad phylogenetic distribution among the subclades within clade I. Interestingly, these fruit strains had closely related strains from other ecological niches in India, including those from patients, hospital environments, and marine environments. However, strains from other parts of the world that are genetically similar to the Indian fruit strains have also been found, including one from France and two from Canada. Interestingly, the French strain (CNRMA15-337; SRR10723348) was from a patient on Reunion Island in the Indian Ocean and who had travel history to India (6, 34). Similarly, of the two strains from Canada that showed close relationships to three fruit strains (VPCI/F1/A/2020, VPCI/F5/A/2020, and VPCI/F6/E/2020), one (strain CNISP2, i.e., SRR10554762) was from a patient who was previously hospitalized in India (35), while no demographic information is available for the other strain, B13464 (i.e., SRR10461158) (6). Together, these results suggest that stored apples could be a source of transmission of C. auris in health care settings. Indeed, more extensive sampling of other stored fruits and from other geographic and ecological niches could reveal additional strains showing close relationships among the ecological niches.

Aside from C. auris, this study also revealed other pathogenic *Candida* spp. such as C. guilliermondii (27%), C. parapsilosis (23%), and C. tropicalis (19%), on the surfaces of fruits. Indeed, among the 22 yeast species isolated from fruits, 12 are known to be capable of causing invasive fungal infections. A recent report showed that Candida blankii caused an outbreak of nosocomial fungemia in a neonatal intensive care unit in India (36). At present, the source(s) of the pathogen for the outbreak is not known. However, the isolation of C. blankii from apple surface suggests that apples could serve as a contact point for transmission of this yeast to patients. Detailed genotypic analyses of strains from diverse sources are needed in order to identify the potential sources for this and other outbreaks.

This study expands our understanding of C. auris ecological niches and will serve as a resource for future studies exploring this yeast in natural environments. Our study revealed that the ecology of C. auris is likely more complex and diverse than what is known so far. In addition, our study suggested that fungicide application on stored apples and potentially other fruits could be a significant selective force for drug resistance in clinics. Together, the information generated here could guide future epidemiological studies of C. auris in other parts of the world and help design better management and control strategies against this important fungal pathogen.

## MATERIALS AND METHODS

### Isolation and identification of yeasts from fruits.

A total of 84 fruits were processed within a day of receiving. Briefly, sterile swabs were swept over the epicarp of fruits and inoculated on Sabouraud dextrose agar with chloramphenicol and gentamicin (SDA-CG) plates, on CHROMagar Candida (Becton, Dickinson, Baltimore, MD, USA), and in yeast nitrogen broth (YNB) (2, 11) and incubated at 37°C. Also, a small piece of flesh without epicarp was cut and homogenized in saline. Four hundred microliters of suspension was inoculated on SDA-CG, on CHROMagar Candida, and in YNB (2, 37,). Identification of yeast colonies was done by matrix-assisted laser desorption ionization–time of flight mass spectrometry (MALDI-TOF MS; Bruker Biotyper OC version 3.1, Daltonics, Bremen, Germany) and internal transcribed spacer region (ITS) sequencing (2).

### Detection of fungicides in the apples using GC-MS and LC-MS/MS.

Extraction and cleanup of unpeeled whole apples were done as recommended by pesticide residue analysis manual 2007, ICAR (38). Briefly, gas chromatograph (Nexis 2030, Shimadzu, Japan) with mass selective triple quadruple detector (GC-MS/MS TQ 8040 NX, M/s Shimadzu, Japan) and liquid chromatography (Aquity UPLC, Waters Corporation, USA) fitted with mass detector (AB 3200, AB Sciex, USA) was used for analysis. For separation of analytes through GC-MS and LC-MS/MS, SH-Rxi-5Sil MS and Chromolith RP–18 columns were used, respectively (39).

### Morphological characterization of Candida auris.

CasSA and reference B8441 strains were subcultured on SDA, yeast peptone dextrose agar (YPD), and rice Tween agar and incubated at 28°C, 37°C, and 42°C for 5 days. Cells were monitored under optical microscope (Nikon H600L, Japan) at ×40 magnification.

**Growth kinetics.** For calcofluor white (CFW) susceptibility and salt tolerance testing, all CasSA along with B8441 were grown at 5 mg/L to 2,560 mg/L concentrations of CFW and 10% of sodium chloride (NaCl). Growth turbidity was measured by microplate reader (Infinite 200 Pro, Tecan, Switzerland) at 37°C for 24 h as described previously (7). Also, growth at 37°C and 42°C for 48 h was checked as detailed previously (7).

**Determination of ploidy of C. auris strains.** Two CasSA strains along with clade I reference strains (B11098 and B8441) and Candida glabrata (ATCC 15545) were subjected to fluorescence-activated cell sorting (FACS; FACSAria III, BD Biosciences, USA). Sample preparation and analysis were done as recommended by Todd et al. (40). Flowjo 7.8 software was used to interpret the FACS results.

### Antifungal susceptibility testing against medical antifungals and agriculture azoles.

Antifungal susceptibility testing (AFST) was performed using the CLSI broth microdilution method (BMD), following M27-A3 (41). AFST for 10 antifungals was performed as described previously (14). Agriculture triazole fungicides tested were tebuconazole (TEB), epoxiconazole (EPX) propiconazole (PCZ), bromuconazole (BRO), flusilazole (FLU), and two diazoles, namely, pyraclostrobin (PCL) and carbendazim (CBZ). All agriculture azoles were procured from Sigma (St. Louis, MO, USA), and dilutions for TEB, EPX, PCZ, PCL, BRO, and CBZ were 0.25 to 128 mg/L and 0.125 to 64 mg/L for FLU. Candida krusei ATCC 6258 and Candida parapsilosis ATCC 22019 were used as quality control strains. All statistical parameters were calculated by using Prism version 6.00 (GraphPad Software).

### Genome sequencing.

DNA extraction was done by QIAamp DNA minikit as described previously (2, 7). Whole-genome sequencing (WGS) libraries were prepared using NEBNext ultra II DNA FS kit (New England Biolabs, Ipswich, MA, USA) and sequenced on Illumina HiSeq 4000.

**Variant identification and phylogenetic analysis.** For phylogenetic analysis, genomes of 42 previously published Indian strains, comprising 24 clinical strains, 5 strains from hospital environments, and 13 marine environmental strains from Andaman Islands, India (2, 4, 7), were retrieved for comparison along with B8441, the clade I reference strain. All strains were subjected to NASP pipeline for genome sequencing analysis. First, raw reads with average quality value of 5 bp window lower than 20 or length shorter than 80 were removed using Trimmomatic v.0. Then, the filtered reads were mapped to reference genome B8441 using BWA mem v0.7 and variants were identified using GenomeAnalysisTK v2.7.4 (3, 7). Afterwards, single nucleotide polymorphisms (SNPs) were filtered out if they were in reference genome duplicated regions, failed the minimum coverage threshold of 10, or had less than 90% of the reads supporting the call. The phylogenetic tree was constructed using RAxML v8.0.25 (42) under ASC_GTRCAT nucleotide substitution model and 1,000 bootstrap replicates. After we removed SNP sites that had ambiguous calls in over 0.5% of the samples, 1,281 SNP sites remained among the Indian samples and were concatenated. Additional phylogenetic analysis used B8441 as reference and includes 16 fruit isolates as well as 487 previously reported clade I C. auris samples. In this broad analysis, SNP sites with ambiguous calls in over 0.5% of the samples were removed and the remaining SNPs were concatenated for each sample. Maximum-likelihood phylogeny was constructed using RAxML-HPC2 on XSEDE in the CIPRES Science Gateway (43). The tree uses the ASC_GTRCAT nucleotide substitution model and 1,000 bootstrap iterations and was visualized with iTOL (44).

**Divergence time estimation of fruit isolates.** The sequences of genes that contain SNPs in any of the 59 samples (43 previously published genomes and 16 CasSA) were concatenated and aligned as one partition. The length of MCMC chain was set to 100 million steps, and samples were recorded every 5,000 steps. Tracer v.1.7.1 was used to investigate MCMC convergence (2). The MCMC chain converged with the effective sample sizes exceeded 700. A maximum clade credibility tree was generated by TreeAnnotator v1.8.4 after discarding 5% as burn-in and visualized in FigTree v1.4.4 (http://tree.bio.ed.ac.uk/software/figtree/). The emerging dates of the fruit isolates were estimated by calculating time to the most recent common ancestor of other Indian isolates.

### Detection of cuticular wax components in apples using GC-MS/MS.

A total of five apples, including two C. auris-positive and three C. auris-negative apples representing stored (*n* = 3) and freshly picked (*n* = 2) from organic orchards, were screened for wax profiling. Apple peel was removed and grounded and the extraction of organic components was done as described previously (39). For wax profiling, gas chromatography–tandem mass spectrometry (GC–MS/MS; Agilent 7890A, USA) with an HP-5 column coupled with an Agilent 7000 QQQ MS was used. Analysis of samples was done as recommended by Bhatnagar et al. (45). Metabolite identification was done by comparing their mass spectra with those obtained from authentic samples and/or the NIST (National Institute of Standards and Technology, USA) mass spectral database using Mass-Hunter software (version B. 05.00).

### Metagenomics.

Peels were removed and placed in 0.05 M phosphate buffer (pH 6.8) for 30 min with shaking at 200 rpm. Peels were discarded and the solution was stored at −20°C until subsequent analysis. Microbial genomic DNA was extracted by using a column-based method with a QIAamp DNA minikit (Qiagen, Hilden, Germany) and quantified by QUBIT 3 Fluorometer using dS DNA HS dye. Primers ITS1 and ITS2 were used to amplify and sequence the ITS1 region. The processed paired-end reads were mapped using KMA (46). The KMA result file was subjected to metagenomic classification using CCmetagen v.1.2.5 and Kraken2 v.2.1.1, and the report was visualized using krona (CCMetagen-1.2) and Pavian (47–49).

### Data availability.

The genome sequences of all 16 C. auris strains isolated in the present study from the surfaces of apples are accessible through BioProject number PRJNA809768.
