# Supplementary material for: Candida auris on Apples: Diversity and Clinical Significance
Source: mBio. 2022 Mar 31;13(2):e00518-22. doi: 10.1128/mbio.00518-22 (PMC9040835; doi:10.1128/mbio.00518-22)
Supplement: FIG S1 [file mbio.00518-22-sf001.docx]

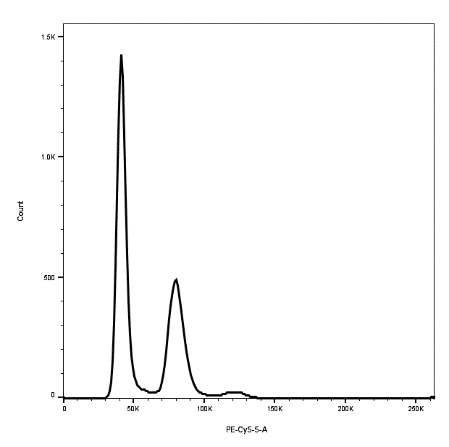


**A.** *Candida glabrata* (ATCC 15545)


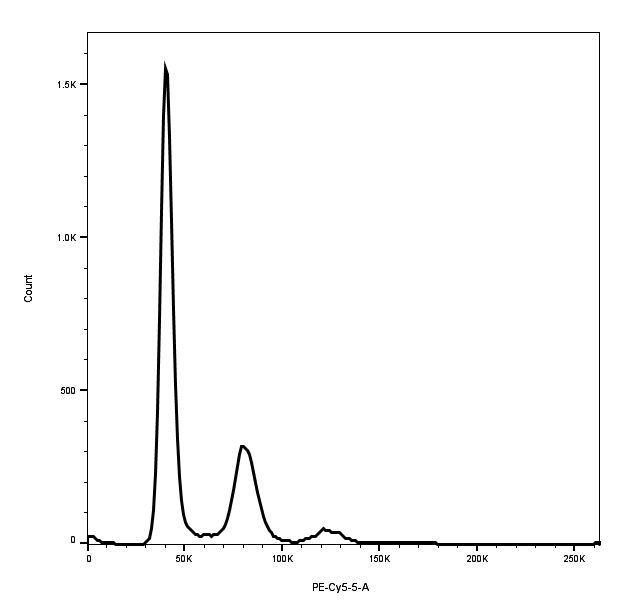


**B1.** B11098 – Clade I


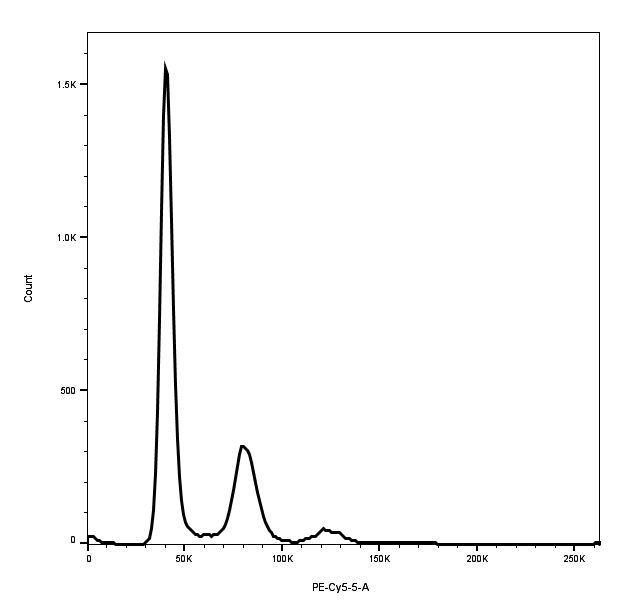


**B2.** B8441 – Clade I


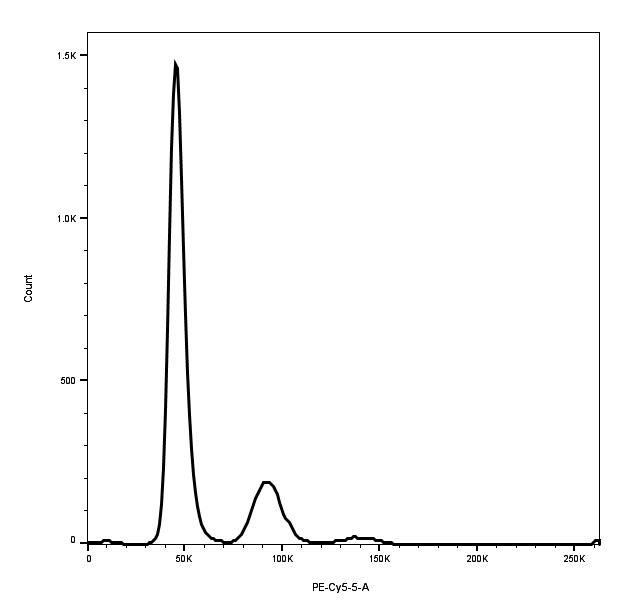


**C1.** VPCI/F6/A/2020


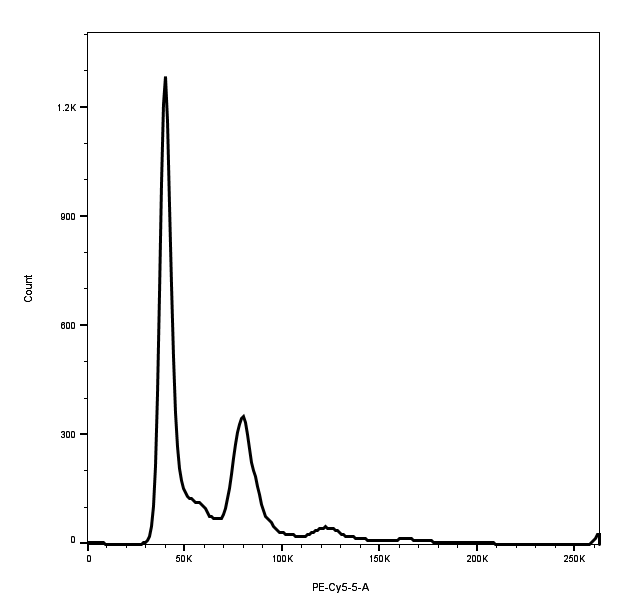


**C2.** VPCI/F1/A/2020

**Fig. S1**: Histograms representing DNA content obtained by FACS. Fig.B1-2 show haploid *C.auris* reference clade I strains (B11098, B8441). Fig.C1-2 show haploid *C.auris* strains on the surfaces of apples (*Cas*SA). Fig. A show a haploid *C. glabrata* strain (ATCC 15545) for comparison, was also analyzed. The X-axis represents nuclear fluorescence, and Y-axis represents cell number.
