# Supplementary material for: Candida auris on Apples: Diversity and Clinical Significance
Source: mBio. 2022 Mar 31;13(2):e00518-22. doi: 10.1128/mbio.00518-22 (PMC9040835; doi:10.1128/mbio.00518-22)
Supplement: TABLE S2 [file mbio.00518-22-st002.docx]

**Table-S2:** Characterisation of aliphatic components in five apples including two *C. auris*-positive and three *C. auris*-negative apples representing stored (n=3) and freshly picked (n=2) from organic orchards.

| **Metabolites classes Code** | **Compounds** |
| --- | --- |
| **Alkanes (AL)** | |
| AL1 | α Farnesene (C15) |
| AL2 | Tetradecane (C14) |
| AL3 | Pentadecane (C19) |
| AL4 | Octadecane (C18) |
| AL5 | Hexadecane, 2,6,10,14-tetramethyl (C19) |
| AL6 | Heptadecane (C21) |
| AL7 | Anthracene (C17) |
| AL8 | 3-(4'-hydroxyphenyl)-2,2,4-trimethylpentane |
| AL9 | Pentatriacontane |
| AL10 | Phenanthrene (C16) |
| AL11 | Tricosane (C23) |
| AL12 | Nonadecane (C19) |
| AL13 | Dodecane (C15) |
| AL14 | Tetracosane |
| AL15 | Eicosane (C20) |
| **Fatty acids (FA)** | |
| FA1 | Phthalic acid |
| FA2 | Palmitic acid/ Hexadecanoic acid (C16:0) |
| FA3 | Cyclopropanecarboxylic acid |
| FA4 | Docosanoic acid |
| FA5 | (3,5-Dimethylphenyl) carbamic acid |
| FA6 | Lauric acid / Dodecanoic acid |
| FA7 | Phosphoric acid |
| FA8 | Caproleic acid |
| FA9 | Oleic acid (C18:1) |
| FA10 | Stearic acid/Octadecanoic acid (C18:0) |
| FA11 | Propanoic Acid |
| FA12 | Hexanoic acid |
| FA13 | Carbonic Acid |
| FA14 | Linoleic Acid |
| **Ketones (K)** | |
| K1 | (Z)-3-(Hex-3-enyl)cyclopent-2-en-1-one |
| K2 | 2,3,6-Trimethylphenylbutan-2-one |
| K3 | 6,9-dimethyl-7-methoxynaphtho(1,8-bc)pyran-3(2H)-one |
| K4 | 2-Decanone |
| K5 | 5-Methyltricyclo(5.3.1.1(3,9))dodecane-2,8-dione |
| **Alcohols (A)** | |
| A1 | 2-(2,4,4-Trimethylpent-2-yl)phenol |
| A2 | 4-Nonylphenol |
| A3 | Phenol |
| **Esters (E)** | |
| E1 | Benzyl benzoate |
| E2 | Methyl linoleate |
| E3 | butyl 2-methylpropyl ester |
