# Supplementary material for: Candida auris on Apples: Diversity and Clinical Significance
Source: mBio. 2022 Mar 31;13(2):e00518-22. doi: 10.1128/mbio.00518-22 (PMC9040835; doi:10.1128/mbio.00518-22)
Supplement: TABLE S3 [file mbio.00518-22-st003.docx]

**Table S3:** Taxonomic classiﬁcation of fungal species detected from the surface of freshly picked apple 1 from organic orchards.

| Name | ITS1 counts | Percentage of counts |
| --- | --- | --- |
| *Schizosaccharomyces pombe* | 2077862 | 97.8% |
| *Sugiyamaella lignohabitans* | 10858 | 0.5% |
| *Zymoseptoria tritici* | 9292 | 0.4% |
| *Colletotrichum higginsianum* | 8822 | 0.4% |
| *Neurospora crassa* | 2773 | 0.13% |
| *Fusarium oxysporum* | 2451 | 0.11% |
| *Candida glabrata* | 2360 | 0.11% |
| *Sporisorium graminicola* | 1877 | 0.08% |
| *Aspergillus oryzae* | 1476 | 0.06% |
| *Pichia kudriavzevii* | 1454 | 0.06% |
| *Pyricularia grisea* | 956 | 0.04% |
| *Candida orthopsilosis* | 807 | 0.03% |
| *Eremothecium sinecaudum* | 804 | 0.03% |
| *Drechmeria coniospora* | 802 | 0.03% |
| *Kluyveromyces lactis* | 349 | 0.01% |
| *Pyricularia oryzae* | 229 | 0.01% |
| *Eremothecium cymbalariae* | 195 | 0.009% |
| *Fusarium venenatum* | 177 | 0.008% |
| *Malassezia restricta* | 82 | 0.003% |
| *Fusarium graminearum* | 54 | 0.002% |
| *Botrytis cinerea* | 40 | 0.001% |
| *Tetrapisispora phaﬃi* | 33 | 0.001% |
| *Talaromyces rugulosus* | 16 | 0.0007% |
| *Candida dubliniensis* | 16 | 0.0007% |
| *Cryptococcus neoformans* | 15 | 0.0007% |
| *Cryptococcus gattii VGI* | 12 | 0.0005% |
| *Thermothelomyces thermophilus* | 10 | 0.0004% |
| *Candida albicans* | 6 | 0.0002% |
| *Fusarium verticillioides* | 5 | 0.0002% |
| *Fusarium fujikuroi* | 3 | 0.0001% |
| *Thermothielavioides terrestris* | 3 | 0.0001% |
| *Aspergillus fumigatus* | 3 | 0.0001% |
| *Ogataea parapolymorpha* | 3 | 0.0001% |
| *Naumovozyma dairenensis* | 2 | 0.00009% |
| *Eremothecium gossypii* | 1 | 0.00004% |
| *Kazachstania africana* | 1 | 0.00004% |
| *Brettanomyces nanus* | 1 | 0.00004% |
| *Scheﬀersomyces stipitis* | 1 | 0.00004% |
