# Supplementary material for: Candida auris on Apples: Diversity and Clinical Significance
Source: mBio. 2022 Mar 31;13(2):e00518-22. doi: 10.1128/mbio.00518-22 (PMC9040835; doi:10.1128/mbio.00518-22)
Supplement: TABLE S4 [file mbio.00518-22-st004.docx]

## Table S4: Taxonomic classiﬁcation of fungal species detected from the surface of freshly picked apple 2 from organic orchards.

| Name | ITS1 counts | Percentage of counts |
| --- | --- | --- |
| *Schizosaccharomyces pombe* | 953147 | 85% |
| *Aspergillus oryzae* | 41215 | 3.6% |
| *Sugiyamaella lignohabitans* | 27146 | 2.4% |
| *Colletotrichum higginsianum* | 18442 | 1.6% |
| *Zymoseptoria tritici* | 13266 | 1.1% |
| *Candida glabrata* | 12468 | 1.1% |
| *Eremothecium sinecaudum* | 10413 | 0.9% |
| *Drechmeria coniospora* | 7527 | 0.6% |
| *Neurospora crassa* | 7168 | 0.6% |
| *Sporisorium graminicola* | 6743 | 0.6% |
| *Pichia kudriavzevii* | 5985 | 0.5% |
| *Eremothecium cymbalariae* | 4004 | 0.3% |
| *Fusarium oxysporum* | 3506 | 0.3% |
| *Candida dubliniensis* | 1399 | 0.1% |
| *Talaromyces rugulosus* | 1005 | 0.08% |
| *Fusarium venenatum* | 944 | 0.08% |
| *Pyricularia grisea* | 898 | 0.08% |
| *Candida albicans* | 857 | 0.07% |
| *Pyricularia oryzae* | 610 | 0.05% |
| *Ogataea parapolymorpha* | 569 | 0.05% |
| *Aspergillus fumigatus* | 511 | 0.04% |
| *Kluyveromyces lactis* | 511 | 0.04% |
| *Malassezia restricta* | 452 | 0.04% |
| *Fusarium graminearum* | 420 | 0.03% |
| *Tetrapisispora phaﬃi* | 414 | 0.03% |
| *Botrytis cinerea* | 196 | 0.01% |
| *Scheﬀersomyces stipitis* | 45 | 0.004% |
| *Candida orthopsilosis* | 37 | 0.003% |
| *Tetrapisispora blattae* | 26 | 0.002% |
| *Cryptococcus neoformans* | 25 | 0.002% |

| *Eremothecium gossypii* | 22 | 0.001% |
| --- | --- | --- |
| *Cryptococcus gattii VGI* | 22 | 0.001% |
| *Thermothelomyces thermophilus* | 15 | 0.001% |
| *Fusarium verticillioides* | 7 | 0.0006% |
| *Torulaspora globosa* | 4 | 0.0003% |
| *Fusarium fujikuroi* | 3 | 0.0002% |
| *Cercospora beticola* | 3 | 0.0002% |
| *Naumovozyma castellii* | 2 | 0.0001% |
| *Naumovozyma dairenensis* | 2 | 0.0001% |
| *Kazachstania africana* | 2 | 0.0001% |
| *Lachancea thermotolerans* | 2 | 0.0001% |
| *Brettanomyces nanus* | 2 | 0.0001% |
| *Thermothielavioides terrestris* | 1 | 0.00008% |
| *Kluyveromyces marxianus* | 1 | 0.00008% |
| *Debaryomyces hansenii* | 1 | 0.00008% |
